# Serum Albumin/Globulin Ratio and Cognitive Function in Americans: A Linear Association

**DOI:** 10.1002/brb3.71015

**Published:** 2025-10-29

**Authors:** Ling Tong, Qin Ye, Jia Zhu, Yuan Wang, Gang Chen, Qinyan Wang

**Affiliations:** ^1^ Nursing Department, Sir Run Run Shaw Hospital Zhejiang University School of Medicine Hangzhou China; ^2^ Department of Laboratory Medicine The Children's Hospital, Zhejiang University School of Medicine, National Clinical Research Center for Child Health, National Children's Regional Medical Center Hangzhou China; ^3^ Affiliated Hospital of Jiangnan University Wuxi China

**Keywords:** AGR ratio, albumin, cognitive impairment, globulin, nutrition

## Abstract

**Background:**

Nutritional status and chronic inflammation play crucial roles in cognitive impairment. The albumin–globulin ratio (AGR) serves as a biomarker for assessing nutrition and inflammation; however, its relationship with cognitive function remains unclear. The objective of this study is to investigate the correlation between them.

**Methods:**

The present study utilized data obtained from the National Health and Nutrition Examination Surveys conducted during the years 2001–2002 and 2011–2014. The relationships between the AGR and cognitive impairment were assessed through the application of weighted logistic regression analysis, smoothed fitted curves, the investigation of threshold effects, and the execution of subgroup analysis. In this study, participants with lower AGR values had a greater incidence of cognitive impairment.

**Results:**

The results of the logistical regression model, following adjustments for all potential influencing factors, demonstrated that the AGR may be associated with an increased incidence of cognitive impairment (OR: 0.550, 95% CI: 0.359, 0.843). This finding indicates that for each unit increase in the AGR within the fourth quartile, the prevalence of cognitive impairment decreased by 45%. Smoothed fitted curves and threshold effects demonstrated a linear relationship between the AGR and cognitive impairment (OR: 0.448, 95% CI: 0.312, 0.642). Subgroup analysis revealed that the association between the AGR and cognitive impairment was not influenced by factors such as gender, education, or hyperlipidemia.

**Conclusions:**

Having a lower AGR may be linked to a higher risk of cognitive impairment.

## Graphical abstract

The serum albumin/globulin ratio (AGR) can serve as a new biomarker for identifying individuals at risk of cognitive decline. AGR is an inexpensive inflammatory marker that is easily obtainable in clinical practice.

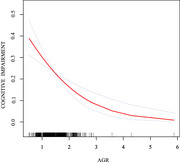

## 1 Introduction

As the global population ages, cognitive impairment (CI) has become a major health concern for older people (Wang et al. 2022). The American Academy of Neurology indicated in 2018 that the mild CI is prevalent in individuals aged over 60 ranges from 6.7% to 25.2%, with the prevalence increasing with age (Petersen et al. 2018). Due to the serious impact of CI on individuals, families, and society as a whole, it has received attention as a public health issue (Pérez Palmer et al. 2022). Currently, there is a lack of definitive strategies to cure CI. However, early detection and timely intervention hold promise for reducing the prevalence of CI (Liu et al. 2020).

There is a broad consensus that this is a more accurate reflection of the inflammatory and nutritional health status of the body (Z. Chen et al. 2022). Previous studies have shown a close association between AGR and muscle mass, cervical cancer prognosis, rheumatoid arthritis, and other conditions (Z. Chen et al. 2022; Oymak et al. 2023; Chen et al. 2021). As the main component of serum proteins, albumin has various biological functions. The free thiol group of albumin imparts antioxidant properties to counteract oxidative stress (Savini et al. 2023). Albumin is also considered a potential marker for evaluating nutritional status. Globulin is composed of various inflammation‐related proteins and is an important indicator of the body's inflammatory response (Marrella et al. 2024). Research has indicated that the inflammatory status and serum albumin concentration are associated with CI (Yang et al. 2024). A study by Gąssowska‐Dobrowolska et al. (2023) revealed that the chronic neuroinflammation observed in Alzheimer's disease patients appears to result from overactivation of glial cells. Researchers have demonstrated it may release various chemicals, including inflammatory cytokines, chemokines, and reactive oxygen species (ROS). This process has been observed to exacerbate neuronal damage. In a further study encompassing 1511 subjects diagnosed with heart failure, low albumin levels were found to be associated with an elevated risk of CI (Zuccalà et al. 2005). Furthermore, reducing inflammation and oxidative stress while increasing nutritional intake is considered a potentially effective strategy for preventing age‐related cognitive decline (Xu et al. 2024; Cutuli et al. 2023; Gurung et al. 2024). As the AGR has been proposed as a marker for assessing the nutritional and inflammatory status of the body, it is speculated that there may be a relationship between AGR and CI. Nevertheless, the relationship between AGR and CI remains to be comprehensively investigated.

Therefore, the present study utilized data from the National Health and Nutrition Examination Surveys, which were conducted from 2001–2002 and 2011–2014 in order to conduct an investigation into the association between the AGR and the occurrence of CI.

## 2 Method

### 2.1 Data Sources and Study Subjects

The NHANES is a research program that is conducted in the United States by the Centers for Disease Control and Prevention (CDC) with the aim of collecting information on the health and nutrition status of the US population. The database received approval from the Institutional Review Board of the National Center for Health Statistics (NCHS), and participants provided written informed consent. The study was conducted in accordance with the Declaration of Helsinki.

**FIGURE 1:**
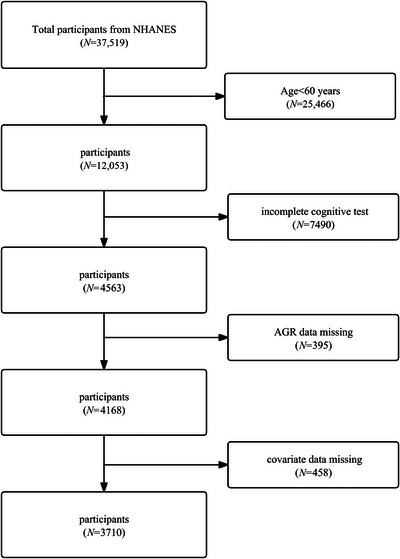
Screening flowchart.

This study utilized NHANES data from 2001–2002 and 2011–2014. A total of 37,519 data entries were initially considered. The following criteria were used to determine the exclusion of individuals: age less than 60 years (*n* = 25,466), missing cognitive data (*n* = 7490), missing AGR data (*n* = 395), and missing covariate data (*n* = 458), including education status, smoking status, marital status, history of hypertension, drinking status, history of high cholesterol, history of diabetes, and serum uric acid (SUA) levels. The final sample size of the study was 3710 (Figure 1).

### 2.2 Study Variables

#### 2.2.1 Cognitive Impairment

The digit symbol substitution test (DSST) is a module of the Wechsler Adult Intelligence Scale (WAIS III) that focuses on performance and is dependent on processing speed, sustained attention, and visual working memory (Peeri et al. 2021). The score is determined by the quantity of correct symbols selected within a time span of 120 s, with a point being awarded for each correctly identified symbol within the stipulated time limit. The maximum possible score is 133. The DSST has been widely used in large‐scale screenings, epidemiological studies, and clinical research (Plassman et al. 2007; Proust‐Lima et al. 2007). However, it should be noted that there is currently no established DSST score definition for CI. Therefore, the present study defines CI as DSST scores below the lowest quartile (DSST < 33) within the study population, a definition that aligns with previous research methodologies (Gong et al. 2021).

### 2.3 Albumin–Globulin Ratio

The measurement of serum albumin (g/dL) and serum globulin (g/dL) was conducted using a DxC800 Synchron analyzer. The AGR is calculated by dividing the albumin concentration by the globulin concentration.

### 2.4 Covariates

The selection of covariates was informed by extant research and encompassed gender, age, education status, race, BMI (body mass index calculated as weight divided by height squared, kg/m^2^), and marital status was categorized as follows: “married/living with partner,” “ widowed/divorced/separated,” or “ never married.” Smoking status was categorized as follows: “ current smoker” (defined as having smoked at least 100 cigarettes in their lifetime) or “ non‐smoker.” Alcohol consumption was categorized as follows: “ ex‐drinker” (defined as consuming at least 12 alcoholic beverages in one year) or “ non‐drinker.” Chronic diseases were assessed through self‐report, with participants being informed by medical professionals about their diagnosed conditions, including hypertension, hypercholesterolemia, and diabetes. Laboratory data included serum urea nitrogen, creatinine, and uric acid.

### 2.5 Statistical Analysis

The weighting variable “wtmec2yr” was selected for the years 2001–2002 and 2011–2014. This finding aligns with the established methodologies employed in previous research endeavors (J. Chen, Liu et al. 2023). Continuous data were represented as the mean ± standard error of the mean, with categorical data presented as 95% confidence interval data. First, we examined the differences between the two groups using weighted chi‐square tests and *t*‐tests. A weighted multivariable logistic model was employed to analyse the independent association between AGR and CI. In the crude model, the adjustment of covariates was not a factor. In the adjusted model, the effects of gender, age, and race were controlled for. In the fully adjusted model, further adjustments were made for age, gender, race, education status, marital status, alcohol consumption, hypertension, smoking status, BMI, hypercholesterolemia, diabetes, serum urea nitrogen, creatinine, and uric acid. Furthermore, in order to assess the linear correlation between AGR and CI, we applied smoothed fitted curves and adjusted for potential confounding factors. We also employed a threshold effect analysis model to investigate the linear correlation between AGR and CI. Finally, we adjusted for variables and conducted subgroup interaction analyses to evaluate heterogeneity in the associations across subgroups. In this study, the threshold for statistical significance was set at *p* < 0.05, and we used weighted methods to reduce the substantial volatility in the dataset.

## 3 Results

### 3.1 The Following Section Outlines the Baseline Characteristics of the Study Population

The present study comprised a total of 3710 participants, including 1815 males and 1895 females. The sample population was divided into two distinct groups, namely those with CI and those without CI, based on the scores obtained from the DSST. The study found that individuals with CI were predominantly older, had attained lower levels of education, consumed alcohol, and exhibited a higher prevalence of hypertension (*p *< 0.05). Interestingly, we found a significantly lower AGR ratio in individuals with CI (*p *< 0.05) (Table 1).

**TABLE 1:** Weighted characteristics of the study population based on cognitive impairment.

	Noncognitive impairment	Cognitive impairment	*p* value
Sex (%)			0.353
Male (*n *= 1815)	45.00 (42.99, 47.03)	47.30 (43.12, 51.52)	
Women (*n* = 1895)	55.00 (52.9, 57.01)	52.70 (48.48, 56.88)	
Age (mean ± SE)	68.85 ± 0.17	73.38 ± 0.36	< 0.001
Age, (%), year			< 0.001
60–69	58.31 (55.86, 60.72)	31.38 (26.97, 36.17)	
70–79	30.23 (28.07, 32.49)	36.18 (32.12, 40.44)	
≥ 80	11.46 (10.20, 12.84)	32.44 (27.59, 37.70)	
Race (%)			< 0.001
Mexican American	2.29 (1.58, 3.32)	8.12 (5.39, 12.04)	
Other Hispanic	2.16 (1.48, 3.13)	11.61 (7.87, 16.81)	
Non‐Hispanic White	85.19 (82.48, 87.55)	58.66 (52.46, 64.61)	
Non‐Hispanic Black	5.85 (4.56, 7.49)	17.90 (14.13, 22.40)	
Other race	4.50 (3.42, 5.91)	3.71 (2.14, 6.37)	
BMI (mean ± SE)	28.77±0.21	28.80 ± 0.33	0.933
BMI (%), kg/m^2^			0.642
< 25	26.44 (23.74, 29.33)	26.65 (22.24, 31.58)	
25–30	38.95 (36.81, 41.15)	41.16 (36.49, 46.00)	
> 30	34.61 (32.17, 37.13)	32.19 (27.12, 37.70)	
Education (%)			< 0.001
Less than ninth grade	3.13 (2.38, 4.11)	29.08 (25.06, 33.45)	
9–11th grade	9.32 (7.83, 11.06)	22.56 (18.62, 27.05)	
High school graduate	23.36 (20.93, 25.99)	24.32 (20.69, 28.36)	
Some college or AA degree	31.38 (28.59, 34.30)	16.77 (13.17, 21.12)	
College graduate or above	32.81 (29.34, 36.48)	7.27 (5.10, 10.27)	
Marital status (%)			< 0.001
Married/living with partner	67.26 (65.12, 69.33)	47.84 (42.60, 53.13)	
Widowed/divorced/separated	29.08 (27.25, 30.97)	45.89 (40.46, 51.43)	
Never married	3.67 (2.86, 4.69)	6.26 (4.11, 9.43)	
Smoking status (%)			0.771
Yes	50.62 (47.65, 53.57)	51.47 (46.61, 56.31)	
No	49.38 (46.43, 52.35)	48.53 (43.69, 53.39)	
Alcohol consumption (%)			< 0.001
Yes	72.38 (69.36, 75.20)	56.85 (53.05, 60.57)	
No	27.62 (24.80, 30.64)	43.15 (39.43, 46.95)	
Hypertension (%)			< 0.001
Yes	54.85 (52.27, 57.41)	69.22 (64.51, 73.57)	
No	45.15 (42.59, 47.73)	30.78 (26.43, 35.49)	
Hypercholesterolemia (%)			0.873
Yes	55.09 (52.36, 57.79)	54.66 (49.51, 59.72)	
No	44.91 (42.21, 47.64)	45.34 (40.28, 50.49)	
Diabetes (%)			< 0.001
Yes	17.25 (15.58, 19.06)	27.82 (24.68, 31.19)	
No	82.75 (80.94, 84.42)	72.18 (68.81, 75.32)	
Urea nitrogen (mean ± SE), mg/dL	16.02 ± 0.13	18.60 ± 0.34	< 0.001
Creatinine (mean ± SE), mg/dL	0.96 ± 0.01	1.23 ± 0.07	< 0.001
Uric acid (mean ± SE), mg/dL	5.60 ± 0.04	5.80 ± 0.07	0.015
AGR (mean ± SE), g/dL	1.56 ± 0.01	1.41 ± 0.02	< 0.001

*Note*: Continuous variables were expressed as mean ± SE, while categorical variables were expressed as 95% CI. BMI stands for body mass index, and AGR represents the ratio of albumin to globulin. *p *< 0.05 was considered statistically significant.

### 3.2 The Following Essay Will Seek to Establish a Correlation Between the AGR and CI

Next, we examined the association between the AGR and CI using weighted logistic regression analysis. In the non‐adjusted model, individuals in the fourth quartile of AGR had a 74% lower prevalence of CI compared to those in the first quartile (OR: 0.260, 95%CI: 0.180, 0.375). After adjusting for all covariates, the association between AGR and CI remained stable. In comparison with those in the lowest quartile of AGR, individuals in the highest quartile demonstrated a 45% lower prevalence of CI (OR: 0.550, 95% CI: 0.359, 0.843), with a *p* value for trend less than 0.001 (Table 2).

**TABLE 2:** The correlation between AGR and cognitive impairment was analyzed using weighted logistic regression.

	Partially adjusted model		Partially adjusted model		Full adjusted model	
	OR (95% CI)	*p* value	OR (95% CI)	*p* value	OR (95%CI)	*p* value
Categories						
Quartile 1	1 (Ref.)		1 (Ref.)		1 (Ref.)	
Quartile 2	0.542 (0.437, 0.672)	< 0.001	0.617 (0.480, 0.794)	< 0.001	0.682 (0.521, 0.893)	0.010
Quartile 3	0.393 (0.311, 0.496)	< 0.001	0.484 (0.371, 0.632)	< 0.001	0.613 (0.434, 0.866)	0.010
Quartile 4	0.260 (0.180, 0.375)	< 0.001	0.395 (0.264, 0.590)	< 0.001	0.550 (0.359, 0.843)	0.011
*p* for trend	< 0.001		< 0.001		< 0.001	

*Note*: Non‐adjusted model: No adjustment for covariates was made. Partially adjusted model: Adjustment was made for gender, age, and race. Fully adjusted model: Adjustment was made for age, gender, race, education status, marital status, smoking status, alcohol consumption, BMI, hypertension, hypercholesterolemia, diabetes, serum urea nitrogen, creatinine, and uric acid. Data are presented as OR (95% CI), *p* values. *p *< 0.05 was considered statistically significant.

Abbreviations: 95% CI, 95% confidence interval; OR, odds ratio.

**FIGURE 2:**
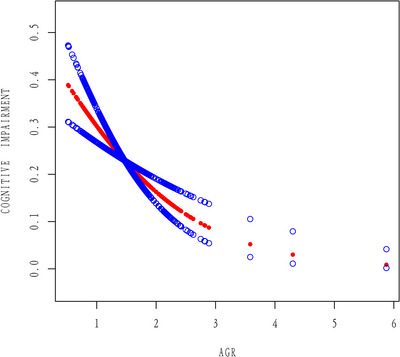
The association between AGR and CI. The red line represents the smoothed curve fit between the variables, while the blue line represents the fitted 95% confidence interval.

Furthermore, on the basis of adjusting for all covariates in the model, A detailed analysis was conducted, with statistical methods such as generalized additive models and smooth curve fitting employed to analyse the data. The present study observed an absence of a nonlinear correlation between AGR and cognitive dysfunction (log likelihood ratio = 0.500); the linear effect was 0.448 (0.312, 0.642) with a significance level of *p* < 0.0001, indicating a linear correlation between AGR and CI (Figure 2 and Table 3).

**TABLE 3:** Threshold effect of AGR on cognitive impairment using full adjusted model.

AGR	Adjusted OR (95% CI)	*p* value
Fitting by standard linear model		
Linear effect	0.448 (0.312, 0.642)	< 0.0001
Fitting by two‐piecewise linear model		
Inflection point(*K*)	1.952	
AGR < 1.952	0.426 (0.290, 0.627)	< 0.0001
AGR > 1.952	0.807 (0.177, 3.688)	0.7820
Log likelihood ratio	0.500	

*Note*: Adjusted for age, gender, race, education status, marital status, smoking status, alcohol consumption, BMI, hypertension, hypercholesterolemia, diabetes, serum urea nitrogen, creatinine, and uric acid. *p *< 0.05 was considered statistically significant.

Abbreviations: 95% CI, 95% confidence interval; OR, odds ratio.

### 3.3 Subgroup Analysis

**FIGURE 3:**
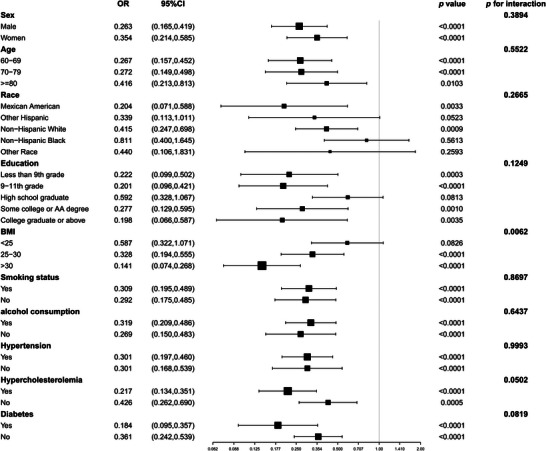
Subgroup analysis of the association between AGR and CI. We adjusted for age, gender, race, education status, marital status, smoking status, alcohol consumption, BMI, hypertension, hypercholesterolemia, diabetes, serum urea nitrogen, creatinine, and uric acid.

Subgroup analysis finally revealed a statistically significant negative relationship between AGR and the occurrence of CI (*p* < 0.05) across gender, age, smoking status, alcohol consumption, and hypertension subgroups. Interaction tests indicated no differences in the association between AGR and CI across subgroups defined by gender, age, race, education, smoking status, alcohol consumption, hypertension, hyperlipidemia, and diabetes (*p* for interaction > 0.05), suggesting that the correlation between AGR and CI is not influenced by these factors. However, we found an interaction effect with BMI (*p* for interaction < 0.05), indicating that the association between AGR and CI exhibited dependency on BMI (Figure 3).

## 4 Discussion

In our cross‐sectional study, which encompassed 3710 participants, we observed a correlation between diminished AGR and an augmented prevalence of CI. This association remained consistent when the data were stratified by gender, age, race, education, smoking status, alcohol consumption, presence of hypertension, hyperlipidaemia, and diabetes.

The relationship between AGR and CI has not been extensively explored. Several other potential factors have been found to be associated with CI in previous studies. A prospective study involving 923 participants investigated the relationship between diet and Alzheimer's disease and reported that the MIND diet and the Mediterranean diet were associated with improved cognitive function (Morris et al. 2015). Randomized clinical trials conducted by Valls‐Pedret et al. (2018) demonstrated that the Mediterranean diet effectively improved cognitive function. These diets may offer neuroprotection by providing adequate nutrients, such as proteins, fats, and vitamins, which can reduce inflammation and oxidative stress in patients with CI (Frisardi et al. 2010). Serum albumin has been used as a biochemical marker for evaluating nutritional status in the body. Some studies have found an independent correlation between reduced serum albumin and decreased cognitive function. For instance, a 6‐year follow‐up study by Schalk et al. (2005) involving 588 elderly individuals identified chronic hypoalbuminemia as a determinant of cognitive decline. A further study on 180 elderly individuals with CI discovered that increasing albumin concentrations led to enhanced cognitive function in patients (Onem et al. 2010). In addition, a trial indicated that albumin replacement through plasmapheresis could delay CI in Alzheimer's disease patients (Boada et al. 2020). Decreased protein levels in late‐stage CI patients may be partly explained by inadequate albumin intake. Research conducted by Qian et al. (2022) discovered that malnutrition can lead to disturbances in thiamine metabolism, affecting the activity and expression of thiamine diphosphate‐dependent enzymes, causing structural damage to glomeruli and renal tubules, and resulting in albumin excretion and microalbuminuria. This may be one of the underlying pathological processes contributing to decreased albumin levels in Alzheimer's disease. Serum albumin is a primary component that maintains plasma colloid osmotic pressure and blood volume. Studies have found associations between blood pressure and other factors with CI (Sabayan and Westendorp 2015). Therefore, a decrease in albumin levels can disrupt the blood flow to the central nervous system, which can affect cognitive function. Furthermore, studies have found that albumin also has inhibitory effects on the formation of amyloid β‐peptide fibrils, thereby improving CI (Milojevic et al. 2007). Therefore, hypoalbuminemia may be a risk factor for CI, while increasing albumin levels may contribute to improved cognitive function.

A growing body of research points to inflammation playing a role in the occurrence and advancement of CI. One study revealed that chronic inflammatory diseases, elevated C‐reactive protein (CRP), and certain inflammatory markers can increase blood‐brain barrier permeability, allowing inflammation to penetrate the brain and increase the incidence of CI (Varnum and Ikezu 2012). Bolós et al. (2017) discovered that the presence of tau protein allows Aβ peptides to induce an inflammatory response, leading to elevated levels of interleukin‐6 (IL‐6), CRP, and tumor necrosis factor‐α. In addition, albumin, which is commonly used to assess nutritional status, is also considered an inflammatory marker (Z. Chen et al. 2022). Previous research has shown that albumin decreases with inflammation regardless of participants' nutritional status (Niedziela et al. 2018). In patients with CI, inflammatory markers such as IL‐6 and CRP increase while albumin levels decrease (Mizrahi et al. 2008). A protective effect in CI has also been found to be associated with high levels of albumin. Studies have shown that albumin possesses antioxidant properties and can promote the synthesis of neurotrophic factors, stimulate neuroglial proliferation, and regulate neuroinflammation (Ralay Ranaivo and Wainwright 2010; Tabernero et al. 2002; Baltanás et al. 2009). However, low albumin levels in patients with CI may disrupt the oxidative/antioxidant balance, further leading to CI (Guo et al. 2011). Moreover, globulin is an indicator of inflammatory conditions and increases during the course of inflammation. Klimkowicz et al. (2004) found that elevated serum globulin is an independent serological marker of cognitive decline in patients with CI. Therefore, it is necessary to consider the effects of both albumin and globulin when assessing nutritional status and inflammatory status in relation to cognitive function. AGR not only reflects nutritional status but also indicates medium‐ to long‐term inflammatory conditions, making it a convenient serological marker (Xie et al. 2021). AGR has been shown to be associated with infectious diseases, cancer prognosis, rheumatoid arthritis, and other conditions (Ye et al. 2020; Yang et al. 2024; Chen et al. 2021). In our study, we observed a greater prevalence of CI in individuals with lower AGR. In a previous cross‐sectional study involving 1827 elderly Japanese individuals, the analysis of patients with CI found a correlation between better cognitive function and higher serum AGR ratio (Maeda et al. 2019). J. Chen, Liu et al. (2021) found that the AGR ratio is increased in healthy young individuals; greater grey matter volume in the olfactory cortex and the parahippocampal gyrus may be a contributing factor. In a previous NHANES cross‐sectional study, using data from 2011–2014 involving over 2700 participants, a nonlinear correlation was found between globulin and CI (Huang et al. 2023). This study only considered the relationship between inflammation and cognitive function, while cognitive dysfunction is closely related to nutrition and inflammatory status. Therefore, both albumin and globulin were included in our study. In another NHANES cross‐sectional study involving over 2700 participants, a nonlinear correlation was found between AGR and CI (Yang et al. 2024). However, our study encompassed all periods with cognitive function data, expanding the sample size, and revealed a linear correlation between AGR and CI. Moreover, in our subgroup analysis, we included more data, such as smoking, alcohol consumption, hypertension, high cholesterol, and diabetes indicators, finding that these subgroups did not affect the association between AGR and CI. Importantly, we observed a dependency of the AGR‐CI association on BMI. Within the population with greater than 30, the negative correlation between AGR and CI is more significant, possibly due to better nutritional status in this group. Some studies suggest that in late life, a higher BMI may provide protection for cognitive function by increasing levels of insulin‐like growth factor‐I (IGF‐1), leptin hormone levels, and estrogen production (Harvey et al. 2006; Singh et al. 2006; Yamamoto and Kato 1993). These studies align with our research findings, suggesting that improved nutritional status may benefit cognitive function. Similarly, a recent longitudinal study on the relationship between BMI and cognitive function in elderly Chinese individuals provided evidence for the “obesity paradox.” A study by J. Chen et al. (2025) selected over 2900 participants aged 60 and above from the CHARLS database and found that a higher BMI had a protective effect on cognitive function. The researchers offered explanations for this paradox, suggesting that obesity or higher BMI in the elderly primarily leads to fat accumulation in the legs. The increase in leg fat was associated with improved glucose metabolism, ultimately reducing the risk of CI. Furthermore, other studies revealed that fat factors secreted by adipocytes, such as leptin and adiponectin, could influence cognitive function through neuroprotective effects (Forny‐Germano et al. 2018; Kim et al. 2020). However, adipose tissue can also produce inflammatory factors like IL‐6 and tumor necrosis factor‐alpha (TNF‐α), which are associated with an increased risk of dementia (Darweesh et al. 2018). Therefore, a higher BMI to some extent provides protection for cognitive function, possibly due to the role of adipocytes. Understanding the specific mechanisms of adipocytes in cognitive function will be a focal point of our future research.

We still do not understand exactly how the AGR and the prevalence of CI are negatively correlated. Based on previous research, we speculate that individuals with CI may not be able to eat sufficiently, leading to malnutrition (Kagansky et al. 2005), which in turn may cause a decrease in serum albumin levels. Inflammatory conditions can also contribute to decreased albumin levels and increased globulin levels. Therefore, AGR may be a potential protective factor or indicator of cognitive function.

The present study has several advantages over previous ones. First, we increased the size of our sample by using the NHANES, which is based on population‐representative sampling data. Second, we included a broader range of covariates such as race, specific level of education, BMI, marital status, renal function, etc. We also conducted a weighted analysis to make the sample more representative. In addition, we observed a linear relationship between AGR and CI. Subgroup analysis was then conducted to assess whether this correlation is influenced by other factors. However, we must also acknowledge the limitations of our research. First, due to the cross‐sectional design of the study, we are unable to discern the mechanisms and causal relationships underlying the association between AGR and CI. Therefore, future basic and prospective research is needed to validate these findings. Second, while we took into account some variables, due to limitations of the database, residual confounding factors, including diet, medication interference, and other blood biomarkers, may be present. In the future, our team plans to incorporate additional confounding variables by collecting clinical data. This includes using food frequency questionnaires to assess overall dietary patterns and specific nutrient intake, as well as measuring inflammatory biomarkers in the blood, such as CRP, IL‐6, and TNF‐α, and metabolic indicators like adiponectin, in order to further validate our findings. Lastly, all participants included in this study were Americans aged greater than or equal to 60 years. Therefore, we will be focusing on investigating whether the association between AGR and CI is applicable to younger populations or other ethnic groups in future research.

## 5 Conclusion

To sum up, our research findings suggest a negative correlation between AGR and the prevalence of CI among individuals aged 60 years and over in the United States. We hypothesize that adequate nutritional intake during younger years may be beneficial in reducing the occurrence of CI. However, further large‐scale prospective studies are needed to validate our research findings.

## Author Contributions

L.T. conceived the research idea, conducted data analysis, and drafted the manuscript. Q.Y., G.C., Y.W., and J.Z. conducted data analysis and reviewed the data. G.C. and Q.W. reviewed and edited the manuscript. All authors have read and agreed to the published version of the manuscript.

## Funding

The authors have nothing to report.

## Ethics Statement

The National Health and Nutrition Examination Surveys protocol was approved by the CDC's National Center for Health Statistics Institutional Research Ethics Review Board.

## Consent

All participants provided written informed consent prior to enrollment.

## Conflicts of Interest

The authors declare no conflicts of interest.

## Peer Review

The peer review history for this article is available at https://publons.com/publon/10.1002/brb3.71015

## Data Availability

The datasets generated during the current study are available without restriction in the National Health and Nutrition Examination Surveys repository (http://www.cdc.gov/nchs/nhanes.htm).
